# Stock delineation of striped snakehead, *Channa striata* using multivariate generalised linear models with otolith shape and chemistry data

**DOI:** 10.1038/s41598-021-87143-9

**Published:** 2021-04-14

**Authors:** Salman Khan, Hayden T. Schilling, Mohammad Afzal Khan, Devendra Kumar Patel, Ben Maslen, Kaish Miyan

**Affiliations:** 1 Section of Fishery Science and Aquaculture, Department of Zoology, Aligarh Muslim University, Aligarh, 202 002 India; 2 Centre for Marine Science & Innovation, UNSW Australia, Sydney, 2052 Australia; 3 Sydney Institute of Marine Science, Chowder Bay Road, Mosman, 2088 Australia; 4 Analytical Chemistry Division, CSIR- Indian Institute of Toxicology Research, Lucknow, 226 001 India; 5 Mark Wainwright Analytical Centre, UNSW Australia, Sydney, 2052 Australia

**Keywords:** Freshwater ecology, Biogeochemistry

## Abstract

Otoliths are commonly used to discriminate between fish stocks, through both elemental composition and otolith shape. Typical studies also have a large number of elemental compositions and shape measures relative to the number of otolith samples, with these measures exhibiting strong mean–variance relationships. These properties make otolith composition and shape data highly suitable for use within a multivariate generalised linear model (MGLM) framework, yet MGLMs have never been applied to otolith data. Here we apply both a traditional distance based permutational multivariate analysis of variance (PERMANOVA) and MGLMs to a case study of striped snakehead (*Channa striata*) in India. We also introduce the Tweedie and gamma distributions as suitable error structures for the MGLMs, drawing similarities to the properties of Biomass data. We demonstrate that otolith elemental data and combined otolith elemental and shape data violate the assumption of homogeneity of variance of PERMANOVA and may give misleading results, while the assumptions of the MGLM with Tweedie and gamma distributions are shown to be satisfied and are appropriate for both otolith shape and elemental composition data. Consistent differences between three groups of *C. striata* were identified using otolith shape, otolith chemistry and a combined otolith shape and chemistry dataset. This suggests that future research should be conducted into whether there are demographic differences between these groups which may influence management considerations. The MGLM method is widely applicable and could be applied to any multivariate otolith shape or elemental composition dataset.

## Introduction

Natural markers such as genetic, elemental or morphological markers can be used as tools to delineate populations or stocks, providing important information for fisheries management^1,2^. Otoliths are a common tool used for stock discrimination and numerous studies have shown the potential of otoliths in addressing research problems related to successful fisheries resource management^3,4^. Both otolith shape and elemental composition have become popular and successful tools in discriminating fish stocks^5–8^.

Differences in the shape of otoliths can help to discriminate between groups of fish that are at least partly separated, inhabiting different environments^5,6,9^. Variations in otolith shape increase with the extent of genetic discreteness or geographic separation^10,11^, although disentangling the physiological and environmental influences is often complicated^12^. Similarly, the elemental composition of otoliths can also be used to distinguish between fish populations^13^. Minor and trace elements laid down within the protein matrix become a permanent record of the chemical characteristics of the environment experienced by the fish^14,15^. While both physiological and environmental factors influence the elemental composition of otoliths^16,17^, if fish inhabit different water masses or environments for a certain period of time they can be differentiated via the elemental composition of their otoliths^18–21^. By combining both otolith shape and chemistry data in the same analysis, the ability to differentiate groups of fish can sometimes be improved^22^.

Both otolith shape and otolith chemistry data are usually multivariate with hypothesis testing traditionally conducted using distance-based methods (eg. permutational multivariate analysis of variance (PERMANOVA)^4,23,24^) or model-based methods which assume a gaussian error distribution (eg. multivariate analysis of variance or linear discriminant analysis^25^). Ecologists typically also use these distance-based methods to form ordination plots to visualise the multivariate groupings in a low-dimensional plot (e.g. non-metric multidimensional scaling (nMDS) plots or Canonical Discriminant Analysis^26,27^). The issue, however, with taking these approaches is that they assume homogeneity, with no mean–variance relationship being taken into account in either the hypothesis testing and visualisation techniques. This is concerning for the otolith shape and chemistry data which have strong mean–variance relationships, where the variance increases with the mean concentration and shape parameter value. The otolith data have a natural boundary at zero which creates a mean–variance relationship as observations found away from this boundary become more variable. Particularly concerning is that both the otolith chemistry and shape data have very small values particularly close to this boundary, with the majority of observations being less than 1 making this mean–variance relationship quite strong. A recent study^28^ found that abundances with means less than 1 cannot reasonably be expected to have their variances stabilised, even with a well-chosen transformation due to the strength of this mean–variance trend. Instead, this trend should be explicitly modelled in the testing and visualisation procedure.

Otolith shape data are positive and continuous and as such can be appropriately modelled using the Gamma distribution (traditionally with a log link) which assumes that the variance increases proportionally to the mean squared. If ${y}_{ij}$ is Gamma distributed then $\left({y}_{ij}\right)=k\theta = {\mu }_{ij},$ $Var\left({y}_{ij}\right)=k{\theta }^{2}=\frac{1}{k}{\mu }_{ij}^{2}$, where $k$ and $\theta$ are shape and scale parameters respectively. Otolith chemistry data, however, are often more nuanced, with a proportion of null observations where the measured concentration of a chemical is below the limit of detection and therefore unable to be quantified in the otoliths, as well as a distribution of positive continuous observations for the otoliths which do have the chemical present. The positive continuous observations will have a mean–variance relationship similar to the shape data, however, a Gamma model will not suffice here as it assumes positive continuous data and therefore will not model the null counts. Ecologists have also used $\mathrm{log}\left(y+1\right)$ transforms for similar data to avoid the logging of null counts, however, this has the same issue outlined in^28^ where the transformation is not handling the mean–variance relationship properly and it also isn’t modelling the null counts in a meaningful way, just lumping them all in as log(1). So, a model is required that takes into account both the large number of null observations as well as the mean–variance relationship exhibited in the present observations.

A solution to this problem lies with the methods currently used to deal with Biomass data. Biomass data have very similar properties to the otolith chemistry data, having a number of null observations where the species was not found to be present and a distribution of positive continuous weight samples for the species that are found to be present. The solution to modelling the Biomass data and consequently the otolith chemistry data is the Tweedie Distribution. The Tweedie distribution's suitability to Biomass data is explained in detail in ^29^, however, is largely due its equivalence to summing a Poisson number of gamma random variables. This allows the null observations to be modelled with the Poisson component and the positive continuous observations with the gamma component. The Tweedie distribution also has a flexible mean–variance relationship. If ${y}_{ij}$ are Tweedie distributed then $E\left({y}_{ij}\right)= {\mu }_{ij},$ $Var\left({y}_{ij}\right)={\phi }_{j}{\mu }_{ij}^{\upsilon }$, where $\nu$ is a power parameter that controls the shape of the distribution and ${\phi }_{j}$ (in the context of our study) is a chemical specific dispersion parameter. The mean–variance relationship is therefore defined by Taylor’s power law^30^, which has been shown to arise under a variety of ecological processes^31^.

For ecological studies using multivariate abundance data such as species abundances, multivariate generalised linear models (MGLMs) are becoming more popular as they allow increased certainty and interpretability of the results, flexibility, and efficiency compared to distance-based methods^32,33^. While MGLMs are now common for abundance data^34^, they are rarely used for other datasets despite the flexibility of the method which allows users to specify model parameters to fit a dataset. Otolith chemistry data and shape data can be easily analysed in an MGLM setting^34,35^, for instance by specifying appropriate mean–variance relationships and error distributions for the data^34^. The use of the Tweedie distribution in an MGLM setting is also discussed in detail in ^35^. Appropriately modelling the mean–variance relationship of data avoids misleading results that can arise in the traditional approaches when their homogeneity assumptions are not met^32^. Assumptions for MGLM models can also be readily checked by plotting Dunn-Smyth residuals, which are randomised quantile residuals that have been shown to be effective at detecting many forms of model misspecification for generalised linear models^36,37^. MGLMs have also been found to have higher power then distance-based methods such as PERMANOVA^32^. Visualisations can also be performed in an MGLM setting using a latent variable factor analysis^38,39^ or by taking a copula approach^40^, neither of which mislead users into mistaking differences in variance with differences in the mean.

Another recent technique used to analyse otolith data is machine learning classification methods, which have become prominent as they are robust to many assumptions that are often hard for traditional methods to satisfy^41^. These methods allow the data to be grouped into different classes, which users can align as their ‘population’ markers. This method, however, fails to provide a means for hypothesis testing nor to easily visualise the differences among the groups.

*Channa striata*, locally known in India as “Dharidar-Sol” or “striped snakehead”, is commercially important in food, ornamental and sport fisheries along with other species of the family Channidae. *C. striata* is one of the main food fishes in Asian countries including India. In the last few years, due to increasing anthropogenic activities, unrestrained harvesting and habitat alterations, the natural stocks of the fish have decreased severely^42^. Consequently, feeding and natural breeding grounds of this economically important fish species have been reduced, which has caused a shrinkage in wild populations^42^. Recent work has shown variation in body morphometrics of *C. striata* between 3 sites within India which suggested the potential for sub-population level variation in demographics which should be further investigated^43^. The present study was carried out with the dual aim of firstly, assessing variation in otolith chemistry and shape between the same groups of *C. striata* in India as^43^ to test for further evidence of regional separation, and secondly, demonstrating the use of MGLMs with otolith chemistry and otolith shape data.

## Methods and materials

### Study species, region, and sample collection

*Channa striata* is native to east and southeast Asia. It is found in India, Pakistan, southern Nepal, Sri Lanka, Bhutan, southern China, Bangladesh, and all the countries of southeast Asia. It is also native to the major western islands of the Malay Archipelago, including Sumatra, Borneo and Java. The species has been introduced to the Philippines, eastern islands of Indonesia, New Caledonia, New Guinea, Fiji, south-eastern Russia and South Korea^44,45^. *C. striata* can be found in many types of slow-moving freshwater habitat, including rivers, ponds, lakes, creeks, canals, flooded rice paddies, swamps, and irrigation reservoirs^46^.

Eighteen *C. striata* were collected from each of three locations. Each site was located on a different major river in northern India with fish collected regularly from each site between October 2017 and November 2018 using cast nets (25 mm mesh) and drag nets (28 mm mesh). The three locations were Narora (27° 30′ N; 78° 25′ E) on the river Ganga, Agra (27.1767° N; 78.0081°E) on the river Yamuna and Lucknow (26° 55′ N; 80° 59′ E) on the river Gomti. A site map can be found in^43^. Identification of the fish was based on the descriptions of ^47,48^. Total length was measured to the nearest mm. Otoliths were extracted using forceps, cleaned in fresh water and stored dry before subsequent shape and chemical analysis. Full details of fish used in this study can be seen in Table S1.

All methods were carried out in accordance with the relevant guidelines and regulations. The target fish species is a commercially exploited common food fish in India; therefore the Committee for the Purpose of Control and Supervision of Experiments on Animals (CPCSEA) 2018, Ministry of Environment, Forests and Climate Change, Government of India, does not require ethical approval to be given for this study.

### Otolith shape

The shape of the otoliths was quantified using wavelet coefficients using R v3.6.0^49^. The R package ‘shapeR’^50^ was used to calculate both Normalized Elliptical Fourier and the discrete wavelet coefficients using photographs of each otolith which create mathematical representations of the otolith outlines. All otoliths were photographed using a light microscope and reflected light with the otolith placed with distal surface facing up on a black background. The procedure followed is fully detailed in^50^ although some photos of otoliths needed manual editing to accurately capture the otolith outlines. Once the photos were captured the outlines of the otoliths were smoothed to remove high frequency pixel noise around the otolith outlines using the *smoothout()* function with 100 iterations. The wavelet method then fitted a series of approximating functions within restricted domains to quantify the outline shapes^51^. The elliptical Fourier method by contrast fitted a number of harmonic functions to capture crenulations and lobes on the edges of the otoliths^3^. Both methods result in coefficients which can be used to quantify the shape. Using 10 wavelets (63 wavelet coefficients), > 99% of otolith shape was explained as opposed to the Elliptical Fourier transformed coefficients which were only able to reproduce 95% of the shape (Fourier transformed results not shown) and we therefore proceeded only with the wavelet analysis.

To visualise the difference in mean shape between the three sites, the mean shape was reconstructed using the mean wavelets for each site and plotted using the ‘*plotWaveletShape*’ function. Wavelet coefficients were standardised for fish length as per^50^ before analysis to test for differences between the three sites.

### Otolith chemistry

To remove any surface contamination, otoliths were soaked in 3% H_2_O_2_ for 5 min and immersed for 5 min in 1% HNO_3_. Otoliths were then flooded with ultra-pure water for 5 min to remove the acid. After decontamination, the otoliths were dried under a laminar flow hood and weighed to the nearest 0.1 mg^19,52^. For analysis, the decontaminated otoliths were dissolved in 10 ml of 37% HNO_3_ and the volume was brought up to 25 ml with Milli Q water. Elemental composition of whole otoliths was analysed using inductively coupled plasma atomic emission spectrometry (ICP–AES; Thermo Electron IRIS Intrepid II XSP DUO). Blank samples were used to correct for background noise in readings. The elements (and detection limits in ppm) measured from the otoliths included: Ca (0.005), Na (0.05), Mg (0.0005), Sr (0.0005), Ba (0.0005), Mn (0.001), Fe (0.005), Pb (0.05), Ni (0.005), Zn (0.005), Cd (0.005), Cr (0.005) and K (0.1). All elements were above minimum detection levels except for 4 Zn samples from the Agra site and 7 Cd samples from the Lucknow site. Internal standards Indium (In) and Gallium (Ga) were added in samples and blanks, which were used to correct for the remaining matrix effect and to compensate for instrument drift. Multi elemental standards were prepared with high purity ICP multi-element standard solution IV certiPUR (NIST SRM) obtained from Merck (Germany) using Milli-Q water and analytical grade 2% v/v HNO_3_ for external calibration. Standards were run every 10 samples. A calibration blank was also prepared in the same procedure. The calibration curve was obtained for five points. The concentration of elements in the sample and blank were calculated and expressed as µg g^−1^ (ppm) on dry weight basis^21,52^. All elemental concentrations were converted from ppm to ratios of element:Calcium (mmol:mol) to control for the size of each analysed otolith.

### Statistics

All analysis and figure generation was performed in R v3.6.0^49^. As the initial goal of the paper was to demonstrate the applicability of Multivariate generalised linear models to otolith chemistry and shape data it is necessary to also show the results of a standard analysis method as a baseline. We choose to use a common distance-based analysis, PERMANOVA with a nMDS ordination plot to visualise the differences. Using the ‘vegan’ R package^53^, we created a dissimilarity matrix using Euclidean distances as is common for otolith datasets using the ‘*vegdist()*’ function^23^. Distance based analyses such as PERMANOVA have an often untested assumption of homogeneity of variance between groups. If this assumption is violated, then results can become misleading with inflated standard errors and confidence intervals leading to a possible increase to the Type 1 error rate. In fact, a google scholar search of “otolith PERMANOVA” for 2018 and 2019 revealed less than 10% of papers reported checking this assumption.

To check this assumption in a PERMANOVA setting a dispersion test using the ‘*betadisper()*’ function can be performed where a significant result (P < 0.05) indicates an unequal variance between groups and therefore a violation of the assumptions of PERMANOVA. If this assumption is satisfied, the typical approach will be to proceed with the PERMANOVA for multivariate differences between our three sites using the ‘*adonis()*’ function. An nMDS ordination plot using the ‘*isoMDS()*’ function from the ‘MASS' R package^54^ based upon the earlier created distance matrix can also be created. However, if the homogeneity assumption is not satisfied, then PERMANOVA nor nMDS would not be recommended to be used for the analysis as we would be unable to do hypothesis testing without potentially getting misleading results.

**Figure 1:**
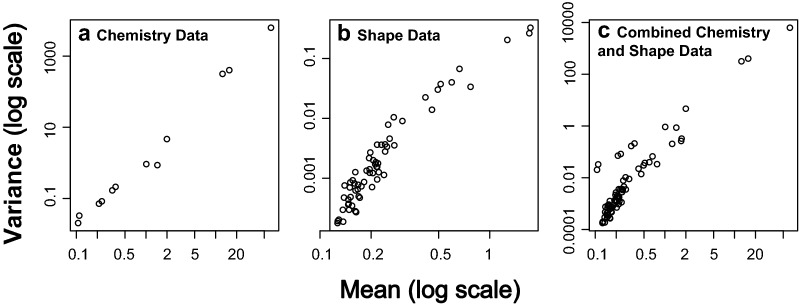
Mean–variance plots showing non-linear relationships for (**a**) the otolith chemistry dataset, (**b**) the otolith shape dataset, and (**c**) the combined otolith chemistry and shape dataset. Note the log scale on both axes.

**Figure 2:**
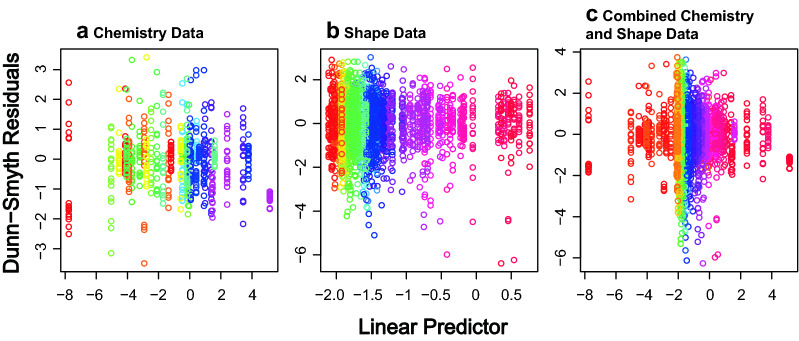
Dunn-Smyth Residual plots for (**a**) the otolith chemistry dataset, (**b**) the otolith shape dataset, and (**c**) the combined otolith chemistry and shape dataset. No strong patterns are visible in any of the subplots, suggesting that our MGLM models were appropriate. Colours show the variables in the analysis.

For the model-based MGLM analysis we followed the analysis guidelines provided in ^33^ following a defined modelling process. We first identified our question: Are there differences in otolith chemistry or otolith shape between the three groups of *C. striata*? Secondly, we considered our data. We had only one predictor variable, Site (a categorical variable), and many response variables (all the elemental concentrations and shape coefficients). Thirdly, we conducted exploratory data analysis but as we only had a single categorical predictor variable this was limited. Next, we selected an appropriate model for the question. Our goal was to compare means between three groups using multivariate data and our *a priori* hypothesis was that there will be multivariate differences between the three sites. Both the otolith chemistry and shape data were positive continuous data but otolith chemistry can contain zeros when elements are below the levels of detection, therefore, Tweedie error distributions were considered as the most appropriate fit for our otolith data while gamma error distributions were considered most appropriate for the shape data. We therefore used multivariate generalised linear models (MGLMs) with a Tweedie error distributions (variance power 1.75, log-link) to test for our hypothesis with chemistry data and a gamma error distribution with log-link for the otolith shape data (coded as a Tweedie distribution with variance power 2 which is equivalent to a gamma distribution). For the combined shape and chemistry data we individually specified error distributions for each variable (Tweedie for the chemistry data and gamma for the shape data). When using multivariate models it is important to understand the relationship between the mean of each response variable and the observed variance^32,55^. To investigate this relationship in our data, we created mean–variance plots which show how the variance changes with the mean of each variable. The mean–variance plots identified that for both chemistry and shape data, as the mean increased, the variance also increased (Fig. 1). As a final step prior to inspecting the results, we assessed our models. To assess if the MGLMs accurately captured the properties of our data, Dunn-Smyth residual plots were inspected for each model. No strong patterns were visible and the models were deemed to be accurately representing our data (Fig. 2), allowing the use of these models to address our hypothesis. All MGLM models were run using the *‘manyany()’* function in the ‘mvabund' R package^34^.

To compare the effectiveness of otolith chemistry and otolith shape data in discriminating the three sites, three MGLMs were run. One only used otolith chemistry data, one only used otolith shape data and one combined both chemistry and shape data. For the two MGLMs involving the elemental data, univariate generalised linear models (GLMs) were also run for each variable to identify which variables were driving the differences. This was conducted using the ‘*manyany()*’ function. The influence of each variable in driving the differences (similar objective to a distance-based SIMPER analysis) was quantified using the individual contribution to the Sum-of-LR^32^, whereby variables with a larger likelihood ratio value are more influential. For the GLMs which included shape data there is no meaningful interpretation of the univariate GLMs as the wavelet shape coefficients cannot be interpreted individually but it does allow the relative contributions of otolith chemistry and shape to be assessed in the combined model. Posthoc tests to identify which sites had showed evidence of differences in specific otolith elemental concentrations were run manually using two sites at a time using the same *‘manyany()’* GLM process and adjusting the *P*-values using the Bonferroni method with the ‘*padjust()*’ function. To visualise the multivariate differences between the 3 fish groups (as an alternative to the commonly applied distance-based ordinations), two factor model-based latent variable ordinations were produced using the ‘boral' R package^39^, again using Tweedie error distributions for chemistry data and gamma error distributions for the shape data with the assumptions being visually assessed^38^. The ‘boral' package takes generalised linear models for each response variable, using Bayesian Markov chain Monte Carlo methods to estimate latent variables that account for between response correlation, which can then be used to visualise multivariate differences on a low-dimension plot^39^. By using generalised linear models, this approach can align the visualisation model with the testing model, check assumptions and specify mean–variance relationships. The code and data used in these analysis is available at: https://github.com/HaydenSchilling/MGLMs-Otoliths

## Results

### Distance-based assumptions and analysis

The dispersion test of equal variance between the samples from the three sites showed that there were significant differences in variance between sites for both the otolith chemistry data (*F*_2,51_ = 9.409, P < 0.001) and the combined chemistry and shape data (*F*_2,51_ = 9.277, P < 0.001). The assumption of equal variance was therefore only satisfied for the shape only dataset (*F*_2,51_ = 0.420, P = 0.659).

For the purpose of our demonstration we proceeded with all three sets of analyses (elemental data, shape data and combined elemental and shape data) but due to the assumption violations caused by the unequal variance between sites, only the shape data analysis should be considered reliable.

**Figure 3:**
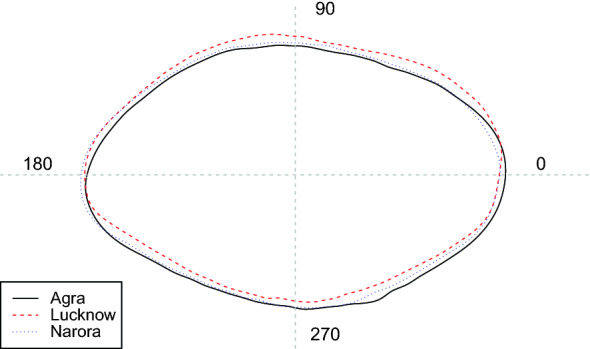
Mean otolith shape from the three sites using the *‘plotWaveletShape’* function in the ShapeR package^50^. The solid black line represents Agra, the dashed red line represents Lucknow and the dotted blue line represents Narora. The wavelet coefficients recreated over 99% of the variance in otolith shape.

**Figure 4:**
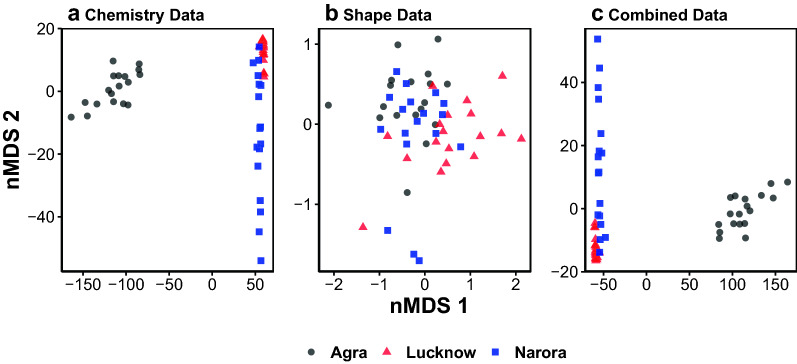
Distance based nMDS ordination based upon (**a**) the otolith chemistry dataset, (**b**) the otolith shape dataset, and (**c**) the combined otolith chemistry and shape dataset. Colours and shapes represent the three groups of *C. striata*. Dissimilarity matrix was based upon Euclidean distances. The assumption of homogeneity of variance was only satisfied for (**b**) Shape Data which would result in the results potentially being unreliable in (**a**) and (**c**).

Using the otolith shape data, the PERMANOVA showed strong evidence of differences between the three sites (*F*_2,53_ = 6.06, P < 0.001, Fig. 3). Visualising the multivariate differences in the nMDS ordination revealed some separation between sites, driven by the Lucknow site while the Agra and Narora samples had considerable overlap (Fig. 4, stress = 0.12).

**Figure 5:**
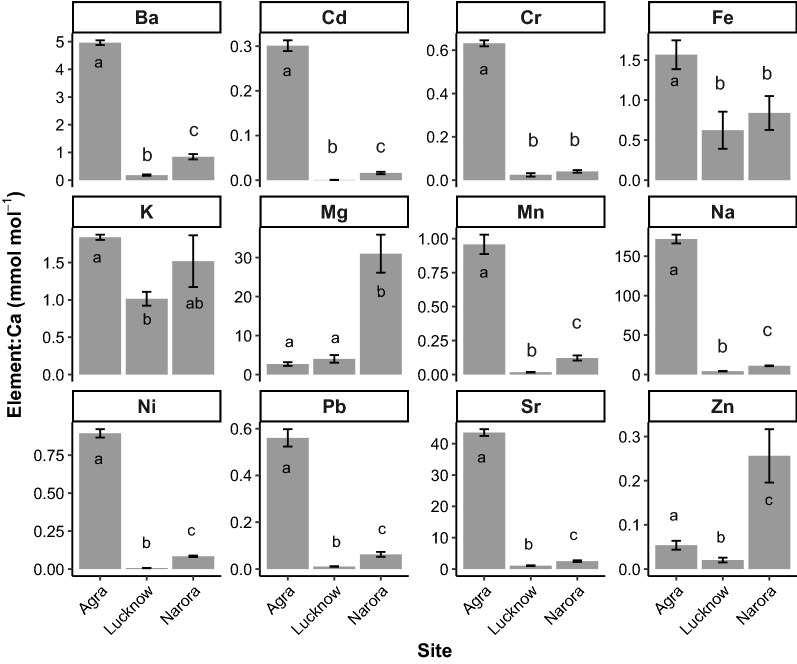
Mean otolith element concentrations (mmol:mol Ca) for each of the three populations. Error bars show 1 standard error. Within a subplot, bars which do not share a common letter are clearly different (MGLM analysis: *P* < 0.05). For univariate GLM results see Table 1.

Using the otolith chemistry data, PERMANOVA showed strong differences between sites (*F*_2,53_ = 513.15, P < 0.001, Fig. 5). Visualisation using the nMDS ordination showed large separation between the Agra site and the other sites along the nMDS 1 axis (Fig. 4, stress = 0.01). The Narora site was heavily dispersed along the nMDS 2 axis with some samples overlapping the Lucknow site.

When combined, the PERMANOVA again showed clear differences between sites (*F*_2,53_ = 511.76, P < 0.001). The separation in the nMDS ordinations was clearly driven by the differences in otolith chemistry with an almost identical pattern observed (Fig. 4, stress = 0.01).

**Figure 6:**
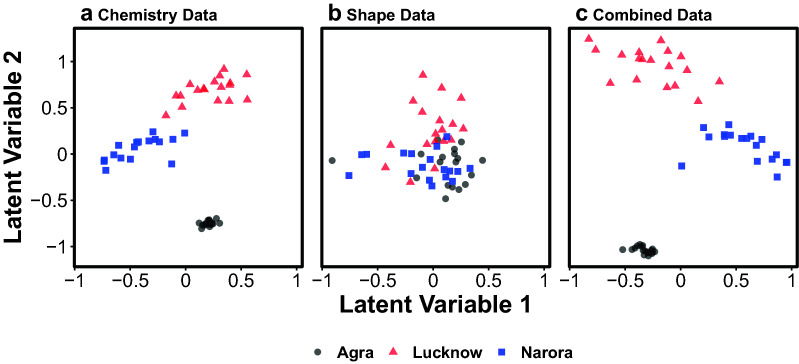
Model-based latent variable ordinations of (**a**) the otolith chemistry dataset, (**b**) the otolith shape dataset, and (**c**) the combined otolith chemistry and shape dataset. Colours and shapes represent the three groups of *C. striata*.

An important point to note in the nMDS plots for the Chemistry only and combined visualisations (Fig. 4) is that the Lucknow group is seen to have points much closer together than the other sites. One could interpret this observation by stating that samples from the Lucknow group are ‘less variable’ then samples from other sites and thus should be easier to distinguish if they didn’t overlap with more variable samples from Norora. This observation, however, is misleading and is a prime example of the dangers of ordination techniques that do not take into account mean–variance relationships (being explained in detail in^32^). Samples from Lucknow had the lowest (or equal lowest) mean concentrations of all chemicals (Fig. 5). This difference in ‘variance’ we are seeing in the nMDS plots for the Lucknow samples is in reality just a difference in mean concentration, with the difference in variance arising from the mean–variance relationship this data has. Without properly accounting for this relationship users can inflate differences in mean concentrations for differences in concentration variability (confounding location and dispersion effects). Conversely, if we look at the model based latent variable ordinations (Fig. 6) that do take into account a mean–variance relationship we not only get ‘greater power’ to pick apart the different populations, but we also have samples from the Lucknow site no longer being depicted with small variability, instead being similar in variability to samples from Norora, removing this previously misleading result.

### MGLM analyses

Using the wavelet coefficients, the MGLM analysis showed clear difference in otolith shape between all three sites (*LR*: 22.368, *P* < 0.001; Fig. 3).

Otolith chemistry was also clearly different between the three sites (*LR* = 1147.9, *P* < 0.001; Fig. 5; Table 1). Large differences in mean concentration were observed for many elements with the Agra site having the highest concentrations of 10 of the 12 tested elements (Fig. 5). The Narora site had the highest concentrations of the other two elements (Zinc and Magnesium; Fig. 5). The Lucknow site showed the lowest concentrations of all elements (Fig. 5).

**Table 1:** Univariate GLM results for the otolith chemistry analysis.

Element	LR	*P*-value	% Contribution
Na	296.57	< 0.001	25.84
Sr	216.61	< 0.001	18.87
Mg	113.60	< 0.001	9.90
Ni	91.59	< 0.001	7.98
Cd	90.53	< 0.001	7.89
Ba	88.85	< 0.001	7.74
Mn	74.82	< 0.001	6.52
Cr	66.78	< 0.001	5.82
Pb	66.60	< 0.001	5.80
Zn	30.30	< 0.001	2.64
Fe	8.23	0.012	0.72
K	3.44	0.016	0.30
Total	1147.92	NA	100

For each element, the Likelihood Ratio test statistic (LR; 2 dp) and *P*-value (3 dp) are shown as well as the % contribution to the multivariate differences (2 dp).

The combined analysis of otolith chemistry and shape also revealed clear differences between all three sites (*LR* = 1166.2, *P* < 0.001). Within this combined analysis most of the differences were driven by the chemistry data (98.4% of the *LR* ratio was made up by the element data).

The differences identified by the MGLMs between sites were visible in the latent variable ordinations (Fig. 6). Similar patterns were visible to those identified in the multivariate generalised linear models with larger differences evident in the otolith chemistry data (Fig. 6a) than the otolith shape data (Fig. 6b). When both datasets were combined, the sites were the most distinct (Fig. 6c).

## Discussion

This study demonstrated how multivariate generalised linear models (MGLMs) can be applied to otolith chemistry and otolith shape data to test for differences between groups of samples. We showed that distance-based analyses including PERMANOVA are not appropriate for our otolith chemistry data due to violations of the assumption of homogeneity of variance stemming from a non-linear mean–variance relationship in the data. This mean–variance relationship can be directly modelled with MGLMs which we then use to show that *Channa striata* from three sites have clear differences in both otolith chemistry and shape. The MGLM method applied here to a simple test between three groups could be easily adapted and expanded to answer other ecological questions requiring more complex model frameworks as is currently done in the broader field of ecology.

### The MGLM method for otolith data

This study has demonstrated the potential for MGLMs to be used as an analysis tool for otolith chemistry and/or otolith shape data, for example in fisheries stock discrimination. We successfully applied a model-based multivariate analysis method to a case study in India and identified differences in otolith chemistry data and otolith shape data for *C. striata* collected from three sites. The MGLM framework which we have used can be considered a robust alternative to the more widely used distance-based analyses including PERMANOVAs. We demonstrated that in some instances such as our example, distance-based analyses are not appropriate due to violations of the assumption of homogeneity of variance. The advantages for using GLMs over distance based methods are well documented in^32^, but briefly we describe the biggest advantages of applying MGLMs to otolith data as well as a potential disadvantage below.

A major advantage of this method is that MGLMs are flexible, being able to specify mean–variance relationships and error distributions that are appropriate to the data, avoiding misleading results from models that do not properly take these relationships into account. These assumptions can be easily checked (and models altered if required) and the appropriateness of the models assessed before any inference is made from the results. We demonstrated this using mean–variance and Dunn-Smyth residual plots in our case study where we demonstrated that the MGLM with Tweedie or gamma error distributions were an appropriate fit to the otolith chemistry and otolith shape data, thus accounting for the non-linear mean–variance relationship (Figs. 1, 2). Not only do MGLMs help to avoid misleading results but they have also been shown to have greater power at detecting effects when compared to traditional distance based approaches^32^. Mean–variance misspecification can also lead to confounding of dispersion and location effects in ordination plots^32^ (which we have verified in this study). This confounding can result in misleading or hard to interpret results when attempting to identify which response the effect is driven by or even a failure to detect multivariate effects unless it expressed in a high variance response. We have also demonstrated the flexibility of MGLMs with our combined shape and chemistry analysis which used different error distributions for the two datasets which ensures both datasets are treated appropriately in the same analysis.

The main downside of using this approach is that computational time can be longer when there are a large number of variables with a Tweedie error distribution. This could be a potential problem for shape data as there are often many coefficients which are used as variables, but with the gamma distribution time is not a concern as the MGLMs with gamma error distributions are faster than with a Tweedie error distribution. Our examples with shape data (gamma error distribution) took only 4 min while the chemistry data (Tweedie error distribution) took 40 min and the combined analysis (combining both Tweedie and gamma error distributions) took 44 min using a single core (8 gb RAM). While these calculations can be run on regular computers the time factor is a trade-off which individual researchers will need to consider, particularly if they do not have access to large computing resources, although with advances in computing software and technology this is likely to become faster and more accessible.

The latent variable model-based ordinations successfully visualised the multivariate differences identified in the MGLMs. The ordinations visually matched the model results with the elemental data clearly driving the separation and the overall separation improving only marginally when shape data was combined with the elemental data. While the current study used a Bayesian model based latent variable method^38,39^, an alternative ordination method directly based upon the MGLM model could be produced using Gaussian copula graphical models which can be run using the ‘ecoCopula’ R package^40^. Both these ordination methods provide an alternative to traditional distance-based ordination methods which we have shown to be misleading by failing to account for mean–variance relations. By following the code provided with this paper, the MGLMs and model-based ordination methods can easily be applied in future studies.

### Implications for *C. striata* in India

Both otolith elemental composition and shape data showed differences between the three sampling sites. Otolith chemistry showed the largest differences while the differences in shape were significant but less clear. The distinct otolith chemistry and shapes suggest that *C. striata* in these three rivers are not regularly mixing. This confirms recent research which used truss morphometry based upon body shape of *C. striata* to suggest that the same three groups analysed in the current study may be distinct sub-populations^43^. Further research should examine key demographic dynamics at each of these three sites including growth rates and age of maturity. If the demographics at each site also differ then management changes may be required^56^.

The unusually high concentrations of some elements in the otoliths likely reflect a heavily polluted environment as in India there continues to be concerns around pollution of waterways^57^. The Yamuna River is very polluted due to many cities lying on its banks and the input of sewage and other industrial effluents directly into the river. For this reason, the Yamuna River is recognised as one of the most polluted in the world^58^. Our fish from the Agra site were located on the Yamuna River and their otoliths are reflective of the heavily polluted state with high concentrations of many elements, particularly heavy metals. It should be noted that fish at the Agra site were also bigger than the other sites (Table S1) but as we used whole otolith elemental composition and controlled for length in the shape analysis, the comparison of differences remains valid as there were very large differences between all three sites, particularly in the elemental composition of the otoliths. There were variations in many elements which contributed to the multivariate differences discussed in the current paper and the drivers behind the specific elemental differences, whether natural or potential pollution present the opportunity for future study.

### Conclusion

This study has successfully demonstrated the use of the Tweedie and gamma error distributions and, by extension, multivariate generalised linear models with otolith data by identifying differences between three sites in India based upon *C. striata* otolith chemistry and otolith shape data. These results suggest that further research into potential demographic differences is now necessary which may then call for the recognition of different stocks of *C. striata*. The MGLM method (and code provided with this paper) is highly flexible and has the potential to be applied to many ecological questions using multivariate otolith data.

## Supplementary Information

Supplementary Information.
