# Supplementary material for: Stock delineation of striped snakehead, Channa striata using multivariate generalised linear models with otolith shape and chemistry data
Source: Sci Rep. 2021 Apr 14;11:8158. doi: 10.1038/s41598-021-87143-9 (PMC8046766; doi:10.1038/s41598-021-87143-9)
Supplement: Supplementary file 1 — Supplementary Information. [file 41598_2021_87143_MOESM1_ESM.pdf]

Supplementary Material to:

Title: Stock delineation of Striped Snakehead, *Channa striata* using multivariate generalised linear models with otolith shape and chemistry data

Authors: Salman Khan[1#], Hayden T. Schilling[2,3#], Mohammad Afzal Khan[1], Devendra Kumar Patel[4], Ben Maslen[5], Kaish Miyan[1]

1. Section of Fishery Science and Aquaculture, Department of Zoology, Aligarh Muslim University, Aligarh -202 002, India
2. Centre for Marine Science & Innovation, UNSW Australia, Sydney 2052, Australia
3. Sydney Institute of Marine Science, Chowder Bay Road, Mosman 2088, Australia
4. Analytical Chemistry Division, CSIR- Indian Institute of Toxicology Research, Lucknow 226 001, India.
5. Mark Wainwright Analytical Centre, UNSW Australia, Sydney 2052, Australia

SK and HTS are joint first authors.

Corresponding author: Hayden T. Schilling, h.schilling@unsw.edu.au , +614 02 9435 4600 (no fax)

Table S1 Details of the samples used in the otolith chemistry and shape analysis of *C. striata*

Site	Sample Size	Mean Fish Total Length (cm)	Minimum Fish Total Length (cm)	Maximum Fish Total Length (cm)	Mean Otolith Weight (mg)	Minimum Otolith weight (mg)	Maximum Otolith weight (mg)
Agra	18	41.0	32.0	48.0	191.1	151.1	264.2
Lucknow	18	32.8	24.5	44.0	107.0	56.1	180.4
Narora	18	31.2	21.5	39.0	103.8	46.2	156.6
